# Diabetes Complications and Cognitive Function in Young Adults with Youth-Onset Type 1 or Type 2 Diabetes: The SEARCH for Diabetes in Youth Study

**DOI:** 10.1155/2023/4860831

**Published:** 2023-04-08

**Authors:** Allison L. B. Shapiro, Anna Bellatorre, Dana Dabelea, Jeanette M. Stafford, Ralph D'Agostino, Amy S. Shah, Elaine M. Urbina, Catherine E. Barrett, Catherine Pihoker, Santica Marcovina, Angela D. Liese, Amy K. Mottl, Elizabeth T. Jensen, Greta Wilkening

**Affiliations:** ^1^Section of Endocrinology, Department of Pediatrics, School of Medicine, University of Colorado Anschutz Medical Campus, Aurora, USA; ^2^Lifecourse Epidemiology of Adiposity and Diabetes (LEAD) Center, University of Colorado Anschutz Medical Campus, Aurora, USA; ^3^Department of Epidemiology, Colorado School of Public Health, University of Colorado Anschutz Medical Campus, Aurora, USA; ^4^Department of Biostatistics and Data Science, Wake Forest University, Winston-Salem, USA; ^5^Department of Pediatrics, Cincinnati Children's Hospital Medical Center, University of Cincinnati, Cincinnati, USA; ^6^Centers for Disease Control, Atlanta, USA; ^7^Department of Pediatrics, University of Washington, Seattle, USA; ^8^Division of Metabolism, Endocrinology and Nutrition, Department of Medicine, University of Washington, Seattle, USA; ^9^Department of Epidemiology and Biostatistics, Arnold School of Public Health, University of South Carolina, Columbia, USA; ^10^University of North Carolina Kidney Center, Division of Nephrology and Hypertension, Department of Medicine, University of North Carolina, Chapel Hill, USA; ^11^Department of Epidemiology and Prevention, Public Health Sciences, Wake Forest University, Winston-Salem, USA

## Abstract

**Methods:**

Using data from the SEARCH for Diabetes in Youth Study cohort, a prospective longitudinal cohort, we examined clustering of complications and their underlying clinical factors with performance on cognitive tests in young adults with youth-onset T1D or T2D. Cognition was assessed via the NIH Toolbox Cognition Battery. The main cognitive variables were age-corrected scores for composite fluid cognition and associated cognitive subdomains. Diabetes complications included retinopathy, microalbuminuria, and peripheral neuropathy (PN). Lipids, systolic blood pressure (SBP), hemoglobin A1c, and other clinical factors were included in the analyses. Clustering was applied separately to each group (T1D = 646; T2D = 165). A three-cluster (C) solution was identified for each diabetes type. Mean values and frequencies of all factors were compared between resulting clusters.

**Results:**

The average age-corrected score for composite fluid cognition differed significantly across clusters for each group (*p* < 0.001). People with T1D and the lowest average fluid cognition scores had the highest frequency of self-reporting at least one episode of hypoglycemia in the year preceding cognitive testing and the highest prevalence of PN. Persons with T2D and the lowest average fluid cognition scores had the highest SBP, the highest central systolic and diastolic blood pressures, and highest prevalence of PN. *Conclusions/Interpretations*. These findings highlight shared (PN) and unique factors (hypoglycemia in T1D; SBP in T2D) that could be targeted to potentially mitigate cognitive issues in young people with youth-onset diabetes.

## 1. Introduction

Diabetes in youth and adults, both type 1 (T1D) and type 2 (T2D), is a significant risk factor for cognitive dysfunction, especially within the executive function subdomains of attention, processing speed, and cognitive flexibility [1–6]. Adults with diabetes-related complications, such as nephropathy, retinopathy, and cardiovascular disease, as well as those with poor glycemic control, are more likely to present with cognitive deficits [7–9]. However, as prior studies have almost exclusively focused on single diabetes complications and their relationship to cognitive outcomes, it remains unknown whether multiple complications and underlying clinical factors co-occur with lower cognitive functioning in discernable patterns.

The influence of diabetes complications, alone or in combination, on cognitive functioning in young individuals with youth-onset diabetes is understudied. Youth and young adults with youth-onset diabetes, T1D or T2D, may be at particularly high risk of poor cognitive outcomes since, in these individuals, diabetes complications occur at a younger age when the brain and cognitive skills are rapidly developing. While severe hypoglycemia [10–12] and diabetic ketoacidosis [13–15] have been consistently shown to independently impact cognitive functioning in youth with diabetes, no studies to date have investigated the association between microvascular complications such as retinopathy, nephropathy, and neuropathy, or macrovascular complications such as major cardiovascular disease, and cognition in youth-onset T1D or T2D. This dearth of data is perhaps owing to the lack of large youth-onset diabetes cohorts where diabetes complications are systematically assessed. Further, it remains unclear whether the co-occurrence of diabetes complications and their influence on poor cognitive outcomes differs by youth-onset diabetes type, T1D vs. T2D.

Investigating the co-occurrence, or clustering, of multiple complications and clinical factors with cognitive outcomes among both T1D and T2D could provide insight into potential shared mechanisms of diabetes complications and cognitive function and may also highlight possible avenues for intervention and treatment of early cognitive dysfunction. Thus, the primary purpose of the present analysis was to examine clustering of diabetes complications, their underlying diabetes clinical factors, and performance on cognitive tests among youth and young adults with youth-onset T1D or T2D who participated in the SEARCH for Diabetes in Youth Study.

## 2. Methods

### 2.1. Participants

The SEARCH for Diabetes in Youth Study is a longitudinal study of individuals with youth-onset (diagnosed <20 years of age) T1D or T2D and has been described extensively in prior publications [3]. In brief, the cohort was recruited from the population-based SEARCH Registry which, since 2002, has continuously enrolled youth-onset T1D and T2D cases from locations in Colorado including Southwestern American Indian reservations, South Carolina, Washington, Ohio, and California [16]. Individuals diagnosed with T1D or T2D in 2002–2006, 2008, and 2012 were seen for a baseline visit shortly after diabetes diagnosis. Two follow-up visits were conducted in 2011–2015 and 2015–2019 among those with ≥5 years diabetes duration. The SEARCH for Diabetes in Youth Cohort Study and Population Based Registry of Diabetes in Youth Study were approved and followed procedures in accordance with the ethical standards of the respective local institutional review boards (COMIRB #01-934). All participants or parent/guardians provided written informed consent and assent, as appropriate by age.

In the current study, we used data from participants who completed in-person procedures from the second follow-up visit (*N* = 1,673) during which the National Institutes of Health Toolbox Cognition Battery was administered when participants were on average 21.6 (SD = 5.1) years old with an average 11.0 (SD = 3.4) years diabetes duration. Additionally, only participants with etiologic-defined T1D (antibody positive, or antibody negative/missing and insulin sensitive; *n* = 1,138) or T2D (antibody negative and insulin resistant; *n* = 301) [17], who were at least 15 years old at the time of the second follow-up visit (T1D = 1,000; T2D = 300), and who had complete data on neurocognitive outcomes and all variables proposed for the cluster analysis were included in the analytic sample (complete case), leaving a sample size of 854 (T1D = 680; T2D = 174).

### 2.2. Demographics, Complications, and Clinical Factors Collected at Second Follow-Up Visit

Participants and parent/guardians completed standardized reporting forms regarding clinical management (e.g., mode of insulin medication and other medications), diabetes-related clinical factors (e.g., self-report hypoglycemic episodes), and well-being (e.g., depressive symptoms) and underwent laboratory testing.

Race and ethnicity, household income, and parents' educational attainment were self-reported. Race and ethnicity were categorized for descriptive purposes into 4 groups: non-Hispanic White (NHW); non-Hispanic Black (NHB); Hispanic or Latino of any race; American Indian or Alaska Native (AIAN) or Asian or Pacific Islander (ASPI) or non-Hispanic other race and ethnicity (NHO). For the cluster analysis, these 4 categories were dichotomized as NHB/Hispanic/AIAN/ASPI/NHO vs. NHW. The highest level of education from either parent was collapsed into a dichotomous variable: high school or less vs. some college or more. Depressive symptoms were assessed using the Center for Epidemiologic Studies Depression Scale (CESD; continuous variable), with higher scores indicating more depressive symptoms and a score greater than 16 indicating risk for clinical depression [18].

Participant height, waist circumference, and weight were measured at baseline and thereafter at each subsequent follow-up visit, and participant waist-for-height ratio (WHtR) area under the curve (AUC) up to the second follow-up visit was derived. Participants self-reported whether they had experienced one or more hypoglycemic or diabetic ketoacidosis (DKA) events (yes/no) over the 12-month period prior to the second follow-up visit. Diabetes duration (years) was derived using the date of diabetes diagnosis and date of the second follow-up visit. Glycemic control was quantified by taking the AUC of repeated laboratory measures of hemoglobin A1c (HbA1c) collected up to the second follow-up visit when cognitive testing was performed (ion exchange high-performance liquid chromatography via Bio-Rad Laboratories, Hercules, CA). Here, we chose to use the AUC of HbA1c rather than a single HbA1c value measured at the time of cognitive testing because the AUC represents the cumulative burden of dysglycemia preceding the cognitive testing, which we believe would be more impactful on overall cognitive functioning, compared to acute effects of dysglycemia at the time of testing.

All blood samples were analyzed by the Northwest Lipid Metabolism and Diabetes Research Laboratories (University of Washington, Seattle). Measurements of total cholesterol (TC), high-density lipoprotein cholesterol (HDL-C), and triglyceride (TG) were performed on a Hitachi 917 autoanalyzer (Boehringer Mannheim Diagnostics, Indianapolis, IN) via enzyme technique. Low-density lipoprotein cholesterol (LDL-C) was calculated by the Friedewald equation where TG concentrations were less than 400 mg/dL (4.52 mmol/L) [19] and by Lipid Research Clinics Beta Quantification [20] where TG concentrations were ≥400 mg/dL (4.52 mmol/L). Very-low-density lipoprotein cholesterol (VLDL-C) was calculated as TG (mg/dL)/5. TG and VLDL-C values were log transformed for use in the cluster analysis due to their right-skewed distributions (descriptive tables show raw values summarized using median and quartiles).

A spot urine sample was collected in the morning at the second follow-up visit. Urine samples were also analyzed by the Northwest Lipid Metabolism and Diabetes Research Laboratories (University of Washington, Seattle). Urine creatinine was measured by the Jaffe method using Roche Diagnostics reagent on the Hitachi 917 autoanalyzer, and urine albumin was measured immunochemically using Dade Behring reagent on a BNII nephelometer. Presence of microalbuminuria, a measure of nephropathy, was defined by the albumin-to-creatinine ratio (ACR) according to the American Diabetes Association guidelines [21]. Specifically, ACR < 30 *μ*g/mg was defined as normal, and ACR 30–299 *μ*g/mg was defined as microalbuminuria.

Diabetes complications measured at the second follow-up visit included retinopathy, peripheral neuropathy (yes/no) [22], and microalbuminuria (ACR ≥ 30; yes/no). Retinopathy was classified using National Health and Nutrition Examination Survey Airlie House/Early Treatment Diabetic Retinopathy Study cutoff values (10–13: none, 14–40: mild, 41–59: moderate, and 60–80: proliferative). Peripheral neuropathy was quantified with the Michigan Neuropathy Screening Instrument [23, 24]. Additional cardiovascular-related clinical factors included central systolic blood pressure (cSBP) and central diastolic blood pressure (cDBP) measured via SphygmoCor (Atcor, PA) and peripheral/brachial systolic blood pressure (SBP).

### 2.3. National Institutes of Health Toolbox Cognition Battery (NIHTB-CB)

As described in detail previously [3], the NIHTB-CB was used to assess cognitive function at the second follow-up visit of the SEARCH Cohort Study. In brief, the NIHTB-CB assesses individual fluid and crystallized cognitive subdomains and generates composite scores for overall fluid cognition and overall crystallized cognition that represents performance across all subdomain tests [25]. Broadly, fluid cognition refers to a set of skills that facilitate a person's ability to learn and problem solve (e.g., processing speed), whereas crystallized cognition refers to information or knowledge that is stored through experiences and interactions with the surrounding environment (e.g., language). Subdomains of fluid cognition included cognitive flexibility (dimensional card sorting test), working (list sorting working memory) and episodic (picture sequence memory) memory, processing speed (pattern comparison speed test), and attention/inhibitory control (flanker inhibitory control and attention test). Subdomains of crystallized cognition obtained in SEARCH included receptive language only, measured via the picture vocabulary test.

All tests were administered to participants on a tablet computer during the second follow-up visit by trained study staff. Completion of all tests took on average 30 minutes. Age-corrected standard scores based upon the normative population were used for the fluid composite score and all subdomain scores. An age-corrected score of 100 (SD = 15) is interpreted as performance equivalent to the national average relative to age-adjusted norms. In the present analysis, our primary cognition measure was composite fluid cognition, as this collectively encompasses the major cognitive skills measured by the NIHTB-CB in SEARCH.

### 2.4. Statistical Analyses

Characteristics of SEARCH participants were described using mean (SD) or median (IQR) for continuous variables and count (%) for categorical variables.

Cluster analysis, a method where observations are grouped according to similarities across multiple variables of interest, was applied to determine the co-occurrence of diabetes complications and clinical factors with cognitive outcomes. Complete-case clustering was conducted separately by diabetes type via Ward's minimum variance method [26], setting an a priori maximum of 5 possible clusters and applying a 5% trim based on low estimated probability densities (resulting analysis subsets: T1D *n* = 646; T2D *n* = 165). Cluster analysis was run without specifying dependence on any single predictor variable. This approach allowed for data-driven grouping of observations based on the underlying similarities of the variables included in the cluster analysis. Variables included were age-corrected cognition scores (composite fluid and subdomains), age at second follow-up visit, sex, race/ethnicity, diabetes duration, CESD score, parental education, WHtR AUC, HbA1c AUC, any self-reported DKA in past 12 months (yes/no), any self-reported hypoglycemic events in past 12 months (yes/no), LDL-C, HDL-C, log-VLDL-C, log-TG, cSBP, cDBP, microalbuminuria (yes/no), peripheral neuropathy (yes/no), peripheral/brachial SBP, and retinopathy (none, mild, or moderate/proliferative). Dichotomous variables having ≤5% prevalence prior to the trim or <4% after were excluded from cluster analyses (i.e., hypoglycemia was excluded from T2D).

Descriptive comparisons across clusters were evaluated using one-way ANOVA or Kruskal–Wallis tests for continuous variables and chi-square or Fisher's exact tests for categorical variables.

## 3. Results

Among the participants included in the analytic sample, those with T1D (*n* = 646) had an average diabetes duration of 11 (3.3) years, were on average 22 (4.2) years old at the second follow-up visit, and predominantly identified as being NHW (63%). Participants with youth-onset T2D (*n* = 165) had an average diabetes duration of 10 (3.6) years, were on average 25 (4.4) years old at the second follow-up visit, and half identified as NHB (50%) (Table 1).

In each of the T1D and T2D groups, a three-cluster solution was identified. In youth and young adults with T1D, individuals in cluster (C) 3 (*n* = 196) presented with composite fluid cognition scores that were, on average, 11 points below the population mean (Table 2; 89.1 (15.3) vs. 100 (15)). Although their scores remained within the normative range, individuals in C3 also scored lower, on average, on tests of processing speed, working and episodic memory, and cognitive flexibility. However, individuals with T1D across all clusters scored, on average, at least 9 points below the population mean on tests of inhibition and attention (81.1 in C3; 82.2 in C2; 90.8 in C1). Youth and young adults with T1D in C3 were more likely to report having had at least one episode of hypoglycemia in the previous 12 months (24.0% vs. 1.5% in C1 and 1.7% in C2). These individuals also had the highest prevalence of peripheral neuropathy (15.8% vs. 0% in C1 and 1.1% in C2).

Individuals with T1D in C2 (*n* = 177) exhibited average composite fluid cognition scores similar to those in C3 but were more likely to present with overall worse diabetes complications and clinical profile. For example, compared to C1 and C3, the C2 group had a higher proportion of individuals who reported having at least one episode of DKA in the previous 12 months (20.9% vs. 9.9% in C1 and 16.3% in C3) and higher prevalence of microalbuminuria (15.8% vs. 0.4% in C1 and 0% in C3) (*p* < 0.001 for each, respectively). Additionally, individuals in C2 had higher HbA1c AUC and LDL-C, VLDL-C, and TGs (*p* < 0.001 for each, respectively). C2 also had greater depressive symptomology as indicated by higher average CESD scores (11.2 vs. 8.2 in C1 and 9.9 in C3; *p* < 0.001).

Individuals with T1D in C1 (*n* = 273), who presented with above average composite fluid cognition and subdomain scores (excepting inhibitory control and attention), presented with the most favorable clinical profile. This included the lowest prevalence of each complication and episodes of DKA and lowest average values for depression, lipids, and measured blood pressures.

Among youth and young adults with T2D, individuals in C3 (*n* = 38) presented with the lowest composite fluid cognition scores (Table 3; *p* < 0.001), which were, on average, 31 points (2 standard deviations) below the population mean (69.2 (11.0) vs. 100 (15)), suggestive of significant cognitive deficits. The C3 group also performed poorly, on average, across all cognitive subdomains, with scores below the population mean by at least one full standard deviation, except episodic memory. Individuals in C2 (*n* = 61) had an average composite fluid cognition score of one standard deviation below the normative mean (84.5 (11.2)), suggestive of mild cognitive deficits, whereas the C1 group (*n* = 66) scored, on average, within the normative range (96.8 (11.8)).

Individuals with T2D in C3 had the highest prevalence of peripheral neuropathy (34.2% vs. 10.6% in C1 and 13.1% in C2). The C3 group also had poor cardiovascular clinical indicators. Specifically, they presented with the highest average cDBP, cSBP, and peripheral/brachial SBP (*p* < 0.05 for each, respectively). Further, compared to C1 and C2, depressive symptomology was greater, on average, in C3, as indicated by higher average CESD scores (16.0 vs. 9.8 in C1 and 12.4 in C2; *p* < 0.01).

Like the T1D cluster results, above, individuals with T2D who were clustered in C2 were more likely to present with an overall worse clinical profile. Individuals clustered in C2 had the poorest glycemic control with higher prevalence of self-reported episodes of DKA in the prior 12 months, and elevated lipids (LDL-C, VLDL-C, TGs), compared to the other clusters. Again, as seen in the T1D results, individuals with T2D who were clustered in C1 with the highest average cognition scores also had the most favorable clinical profile.

## 4. Discussion

We found both shared and unique complications and clinical factors that co-occur with suboptimal cognitive outcomes among a large and diverse cohort of young adults with youth-onset T1D or T2D. Specifically, peripheral neuropathy was found at the highest prevalence within both the T1D and T2D cluster groups who had the lowest overall cognitive test performance, on average, compared to all other cluster groups. These results are consistent with the current but limited literature in middle age and older adults with peripheral neuropathy and T1D or T2D [27–32]. In a recent cross-sectional analysis of the Glycemia Reduction Approaches in Diabetes Study (GRADE) data, Barzilay et al. found significant deficits in episodic memory (immediate recall) and processing speed among adults with peripheral neuropathy and T2D, compared to adults with T2D but without peripheral neuropathy [27]. Among a smaller study of adults with peripheral neuropathy and T1D, Ding et al. also found global cognitive deficits and lower performance on tests of language fluency, attention, and memory, compared to a healthy control group without diabetes [31]. Together, our results and the extant data support peripheral neuropathy as a potentially significant correlate to cognitive deficits in subgroups of people with diabetes regardless of diabetes type and life stage. Unfortunately, all studies to date, including SEARCH, involve cross-sectional analyses of cognition and diabetes-related peripheral neuropathy. Thus, the sequence of events cannot be determined given the current data, and longitudinal, repeated evaluation of cognition and peripheral neuropathy development is needed to draw further insight.

Despite our limited understanding about whether peripheral neuropathy or cognitive dysfunction precedes the other, the clinical implications of their co-occurrence are potentially significant. While the literature remains sparse, studies have shown worse clinical outcomes in peripheral neuropathy, including more frequent and severe foot ulceration [29, 33], among people with lower cognitive functioning and diabetes. These studies are in line with other work demonstrating a strong relationship between cognitive abilities and self-care and treatment adherence in people with diabetes [34–36]. Individuals with cognitive deficits and concurrent peripheral neuropathy may therefore be at greater risk of lower extremity complications such as foot infections, ulcers, and limb amputation secondary to limited self-care practices, compared to individuals with higher cognitive abilities. Thus, considering cognitive testing during initial evaluation and clinical follow-up for peripheral neuropathy may help to facilitate improved resource management for the highest risk patients, regardless of diabetes type.

In our analysis, diabetes type-specific clinical factors were also found to co-occur with lower cognition. Unique to the young adults with T1D, those with worse fluid cognitive performance overall and across all cognitive subdomain tests also had the highest prevalence of self-reporting at least one hypoglycemic episode in the prior 12 months. Our results again align with the extant literature, where hypoglycemia, specifically repeated severe hypoglycemic episodes, is a known correlate of poor cognition among people with T1D across all life stages [37–41]. However, due to the self-report instrument used in SEARCH, we were not able to distinguish between severe (e.g., coma and seizures) and nonsevere episodes of hypoglycemia experienced in the 12 months prior to cognitive testing or investigate co-occurrence of glycemic variability among the participants included in this analysis. Further, while hypoglycemia has also been linked to higher risk of cognitive decline or dementia in older adults with T2D [42, 43], due to low prevalence (<5%) of self-reported hypoglycemic episodes, we were not able to assess hypoglycemia and poor cognition co-occurrence among young adults with T2D in the current analysis.

Young adults with T2D in our analysis were found to have lower fluid cognitive function that co-occurred with worse clinical cardiovascular disease factors such as elevated brachial/peripheral systolic blood pressure and higher central blood pressures. These results, while not previously reported in the youth-onset diabetes literature, are consistent with a large meta-analysis of middle age and older adults (diabetes status unknown) where elevated blood pressure and diagnosed hypertension were associated with cognitive disorders like cognitive impairment or dementia [44]. Some studies in older adults with T2D also report significant associations between high blood pressure and cognitive dysfunction [45, 46], although the data are mixed depending on the age at which high blood pressure developed [47]. Additional studies in adults with T2D have found a significant relationship between lower cognition and elevated central blood pressure measures [48], which are considered robust prognostic indicators of cardiovascular disease. Despite limited information on brain structure and function in people with youth-onset T2D, the effect of hypertension on cognitive function is likely mediated through its impact on the cerebrovascular system including cerebrovascular endothelial dysfunction, inhibited cerebral blood flow (CBF), and microinfarcts in the brain, all of which have been found in adult-onset T2D with hypertension and T2D in one youth study [49–52]. Indeed, cerebrovascular dysfunction in T2D is shown to be involved in suboptimal cognitive and psychiatric health such as worse executive functioning and depression [53]. Given these data in adult-onset T2D, further research is needed to explore the links between blood pressure, cerebrovascular health, mental well-being, and cognitive functioning in young people with youth-onset T2D.

Our cluster analysis also yielded unexpected results such that, among the young adults with T2D, one cluster group (C2) appeared to have attenuated cognitive decrements, relative to the group with the poorest cognitive performance (C3), despite also appearing to have the poorest diabetes control. This unique, and possibly cognitively resilient, group of individuals with T2D demonstrated cognitive performance that was nearly a full standard deviation above the C3 group (poorest cognition). A potentially important distinction between the C2 group and other clusters is the better cardiovascular and obesity outcome. Specifically, the C2 group had the lowest blood pressure profile (peripheral and central blood pressures) and lowest waist-height ratio AUC, on average, compared to both C1 and C3. This is in contrast with the C3 group of young adults with T2D who had the highest blood pressure across all peripheral and central measures, which coincided with the poorest cognitive functioning. Together, these observed differences between the C2 and C3 groups suggest that, in young people with youth-onset T2D, there may be a predominantly vascular contribution to acquiring significant cognitive deficits. This warrants further study.

So too did we see a potentially resilient group among the young adults with T1D. Despite having an average fluid cognitive score suggestive of only mild deficits, young adults with T1D in the C2 group presented with the worst overall clinical profile. This included the highest prevalence of microalbuminuria (16%) and self-report of at least one episode of DKA in the prior 12 months (21%), as well as worse cardiovascular outcomes (e.g., higher BP and cholesterols) relative to the other cluster groups. These findings suggest that factors beyond what were included in the current analysis may contribute to potential cognitive resilience among young adults with T1D (e.g., social support) and additional investigation is needed to draw further conclusions.

Among the significant body of literature focusing on cognition and diabetes complications, we believe that this is the first study to investigate the co-occurrence of multiple diabetes-related complications, their underlying clinical contributors, and cognitive outcomes in youth-onset diabetes. Furthermore, no study has investigated the different patterns of diabetes complications and clinical risk factors between youth-onset T1D and T2D and their co-occurrence with lower cognitive function, as done here. However, the novelty of our results may only be interpreted within the context of this study's limitations. That is, the SEARCH Cohort Study did not collect baseline cognitive data. Thus, we are not able to interpret our results within the context of changing cognitive function due to development of diabetes complications or worsening of underlying clinical factors. Further, as noted before [3], the SEARCH Cohort Study did not collect information about functional outcomes, such as academic performance, that would help to corroborate the level of cognitive impairment reflected by the NIHTB-C scores. Finally, the current analysis did not consider participant medication use, such as blood pressure-lowering, lipid-lowering, or insulin-sensitizing medications. Therefore, our results may only be interpreted independent of the potential positive or negative effects of such medications on cognitive functioning in youth-onset diabetes.

In conclusion, our results provide new evidence of shared and unique overlap of diabetes complications and associated clinical factors to cognitive function in youth-onset T1D and T2D. While replication of these results by other large cohorts is encouraged, our findings should motivate a broader discussion in the field for assessing cognition and tailoring management strategies to address cognitive difficulties and improve outcomes in youth with diabetes.

## Figures and Tables

**Table 1:** Descriptive characteristics of SEARCH participants included in the analytic dataset (*N* = 811).

	Type 1 diabetes (*n* = 646)	Type 2 diabetes (*n* = 165)
Age, years, at second follow-up visit, mean (SD)	21.7 (4.2)	24.5 (4.4)
Sex, female, *n* (%)	315 (48.8)	122 (73.9)

*Race/ethnicity, n (%)*:		
Non-Hispanic White	404 (62.5)	30 (18.2)
Non-Hispanic Black	80 (12.4)	83 (50.3)
Hispanic	130 (20.1)	33 (20.0)
AIAN/ASPI/NHO^1^	32 (5.0)	19 (11.5)

*Parent's highest level of education, n (%)*		
High school graduate or less	119 (18.4)	70 (42.4)
Some college or more	527 (81.6)	95 (57.6)
Diabetes duration, years, mean (SD)	11.1 (3.3)	9.9 (3.6)

^1^AIAN: American Indian or Alaska Native; ASPI: Asian or Pacific Islander; NHO: non-Hispanic other race and ethnicity.

**Table 2:** Results of cluster analysis of cross-sectional data in youth and young adults with type 1 diabetes (*n* = 646).

	Type 1 diabetes		
	C1 (*n* = 273)	C2 (*n* = 177)	C3 (*n* = 196)
*Cognitive function* ^†^			
Composite fluid cognition, mean (SD)	106.6 (11.9)	92.0 (14.7)	**89.1 (15.3)**
Pattern comparison test (processing speed), mean (SD)	109.1 (18.3)	97.1 (20.7)	**95.7 (19.0)**
List sorting working memory test (working memory), mean (SD)	105.9 (11.7)	100.1 (13.6)	**95.7 (14.4)**
Dimensional change card sort test (cognitive flexibility), mean (SD)	107.4 (13.7)	94.7 (15.7)	**92.8 (14.7)**
Picture sequence memory test (episodic memory), mean (SD)	109.4 (16.1)	100.1 (13.9)	**99.2 (17.5)**
Flanker inhibitory control test (inhibition/attention), mean (SD)	90.8 (11.3)	82.2 (11.2)	**81.1 (12.5)**
Picture vocabulary test (receptive language), mean (SD)	112.4 (13.7)	103.6 (11.8)	**97.4 (13.9)**

*Diabetes complications* ^†^			
Mild retinopathy, *n* (%)	107 (39.2)	80 (45.2)	87 (44.4)
Moderate/proliferative retinopathy, *n* (%)	7 (2.6)	12 (6.8)	7 (3.6)
Peripheral neuropathy (yes), *n* (%)	0 (0.0)	2 (1.1)	**31 (15.8)**
Microalbuminuria (yes; ACR ≥ 30), *n* (%)	1 (0.4)	**28 (15.8)**	0 (0.0)

*Clinical factors* ^†^			
Diabetes duration (yrs), mean (SD)	11.3 (3.2)	11.3 (3.2)	10.6 (3.5)
Depressive symptoms (CESD), mean (SD)	8.2 (7.7)	**11.2 (9.9)**	9.9 (7.8)
HbA1c (%) AUC, mean (SD)	8.2 (1.2)	**8.9 (1.4)**	8.6 (1.4)
DKA in past 12 months (yes), *n* (%)	27 (9.9)	**37 (20.9)**	32 (16.3)
Hypoglycemia in past 12 months (yes), *n* (%)	4 (1.5)	3 (1.7)	**47 (24.0)**
LDL cholesterol (mg/dL), mean (SD)	94.8 (23.8)	**117.2 (30.8)**	96.5 (26.2)
VLDL cholesterol (mg/dL), median (IQR)	13.0 (10.0, 17.0)	**21.0 (15.0, 31.0)**	13.0 (10.0, 18.5)
HDL cholesterol (mg/dL), mean (SD)	55.6 (13.2)	**51.7 (12.3)**	56.4 (13.4)
Triglycerides (mg/dL), median (IQR)	66 (51, 86)	**103 (76, 153)**	65 (51, 93)
Waist-to-height ratio AUC, mean (SD)	0.45 (0.04)	**0.48 (0.06)**	0.46 (0.05)
Systolic blood pressure (mmHg), mean (SD)	108.8 (10.0)	**115.1 (9.3)**	105.9 (7.7)
Central systolic blood pressure, mean (SD)	97.0 (9.2)	**103.2 (8.5)**	94.4 (7.6)
Central diastolic blood pressure, mean (SD)	69.6 (8.4)	**77.5 (8.0)**	68.4 (8.1)

CESD = Center for Epidemiologic Studies Depression Scale; AUC = area under the curve; HbA1c = glycated hemoglobin; DKA = diabetic ketoacidosis; LDL = low-density lipoprotein; VLDL = very-low-density lipoprotein; HDL = high-density lipoprotein. ^†^Shading corresponds to the frequency (*n*, (%)) or magnitude (mean/median, (SD/IQR)) of the variable among participants in the respective cluster compared to the other clusters. Variables where the frequency or magnitude was statistically different across the cluster groups are shaded (*p* value <0.05 via chi-square/Fisher's test (categorical) or ANOVA or Kruskal–Wallis test (continuous)). Bold values correspond to the cluster group with worse outcomes.

**Table 3:** Results of cluster analysis of cross-sectional data in youth and young adults with type 2 diabetes (*n* = 165).

	Type 2 diabetes		
	C1 (*n* = 66)	C2 (*n* = 61)	C3 (*n* = 38)
*Cognitive function * ^†^			
Fluid cognitive function, mean (SD)	96.8 (11.8)	84.5 (11.2)	**69.2 (11.0)**
Pattern comparison test (processing speed), mean (SD)	100.7 (19.2)	92.6 (15.1)	**81.1 (20.6)**
List sorting working memory test (working memory), mean (SD)	99.7 (12.4)	90.7 (12.4)	**82.8 (13.2)**
Dimensional change card sort test (cognitive flexibility), mean (SD)	98.6 (14.9)	91.2 (14.2)	**77.8 (12.2)**
Picture sequence memory test (episodic memory), mean (SD)	104.7 (17.4)	95.5 (10.2)	**86.7 (10.4)**
Flanker inhibitory control test (inhibition/attention), mean (SD)	86.6 (10.6)	79.1 (10.2)	**69.0 (9.6)**
Picture vocabulary test (receptive language), mean (SD)	101.6 (14.0)	89.0 (12.7)	**85.8 (12.6)**

*Diabetes complications * ^†^			
Mild retinopathy, *n* (%)	23 (34.8)	26 (42.6)	7 (18.4)
Moderate/proliferative retinopathy, *n* (%)	5 (7.6)	5 (8.2)	3 (7.9)
Peripheral neuropathy (yes), *n* (%)	7 (10.6)	8 (13.1)	**13 (34.2)**
Microalbuminuria (yes; ACR ≥ 30), *n* (%)	13 (19.7)	11 (18.0)	6 (15.8)

*Clinical Factors * ^†,‡^			
Diabetes duration (yrs), mean (SD)	10.4 (3.8)	9.5 (3.4)	9.7 (3.5)
Depressive symptoms (CESD), mean (SD)	9.8 (7.7)	12.4 (8.5)	**16.0 (10.0)**
HbA1c AUC, mean (SD)	7.7 (1.8)	**9.8 (2.1)**	7.6 (2.2)
DKA in past 12 months, *n* (%)	0 (0.0)	**8 (13.1)**	0 (0.0)
LDL cholesterol (mg/dL), mean (SD)	106.2 (38.1)	**116.7 (41.2)**	96.7 (26.1)
VLDL cholesterol (mg/dL), median (IQR)	23.0 (17.0, 35.0)	24.0 (16.0, 34.0)	22.0 (14.0, 30.0)
HDL cholesterol (mg/dL), mean (SD)	**40.3 (9.5)**	45.4 (9.4)	42.7 (9.9)
Triglycerides (mg/dL), median (IQR)	116 (85, 177)	118 (82, 172)	109 (72, 150)
Waist-to-height ratio AUC, mean (SD)	0.64 (0.09)	0.61 (0.09)	**0.66 (0.10)**
Systolic blood pressure (mmHg), mean (SD)	121.2 (13.1)	114.4 (9.2)	**127.4 (14.9)**
Central systolic blood pressure, mean (SD)	108.2 (12.3)	101.5 (8.4)	**113.7 (12.0)**
Central diastolic blood pressure, mean (SD)	77.7 (9.8)	77.0 (8.4)	**82.4 (10.3)**

CESD = Center for Epidemiologic Studies Depression Scale; AUC = area under the curve; HbA1c = glycated hemoglobin; DKA = diabetic ketoacidosis; LDL = low-density lipoprotein; VLDL = very-low-density lipoprotein; HDL = high-density lipoprotein. ^†^Shading corresponds to the frequency (*n*, (%)) or magnitude (mean/median, (SD/IQR)) of the variable among participants in the respective cluster compared to the other clusters. Variables where the frequency or magnitude was statistically different across the cluster groups are shaded (*p* value <0.05 via chi-square/Fisher's test (categorical) or ANOVA or Kruskal–Wallis test (continuous)). Bold values correspond to the cluster group with worse outcomes. ^‡^Self-reported experience of at least one hypoglycemic event in past 12 months not included in cluster analysis due to low prevalence (<5%).

## Data Availability

The data used to support the findings of this study are available on request from the corresponding author and are subject to data use agreements through the SEARCH for Diabetes in Youth Study consortium. The data are not publicly available due to restrictions involving research participant consent.
